# Supplementary material for: Retrospective evaluation of the morphometric properties of intact maxillary sinus using cone-beam computed tomography for sex estimation in an Indian population
Source: PeerJ. 2024 Mar 20;12:e16991. doi: 10.7717/peerj.16991 (PMC10960526; doi:10.7717/peerj.16991)
Supplement: Supplemental Information 2 [file peerj-12-16991-s002.docx]

| **Coordinates of the Curve** | | | |
| --- | --- | --- | --- |
| Test Result Variable(s) | Positive if Greater Than or Equal To^a^ | Sensitivity | 1 - Specificity |
| Length Right | 24.5200 | 1.000 | 1.000 |
|  | 25.8300 | .994 | 1.000 |
|  | 26.4850 | .987 | 1.000 |
|  | 27.2700 | .981 | 1.000 |
|  | 27.7450 | .981 | .994 |
|  | 28.0300 | .981 | .987 |
|  | 28.3150 | .975 | .987 |
|  | 28.3600 | .969 | .987 |
|  | 28.4800 | .969 | .981 |
|  | 28.6150 | .969 | .975 |
|  | 28.6500 | .969 | .969 |
|  | 28.7150 | .969 | .962 |
|  | 28.8050 | .962 | .962 |
|  | 28.8700 | .962 | .956 |
|  | 28.9200 | .962 | .950 |
|  | 28.9850 | .962 | .943 |
|  | 29.1050 | .962 | .937 |
|  | 29.1900 | .962 | .931 |
|  | 29.2200 | .962 | .925 |
|  | 29.2750 | .956 | .925 |
|  | 29.3600 | .956 | .918 |
|  | 29.4450 | .956 | .912 |
|  | 29.4850 | .956 | .899 |
|  | 29.5250 | .950 | .899 |
|  | 29.6100 | .950 | .893 |
|  | 29.6650 | .950 | .874 |
|  | 29.6850 | .950 | .868 |
|  | 29.7200 | .943 | .868 |
|  | 29.7700 | .943 | .862 |
|  | 29.8400 | .943 | .855 |
|  | 29.9100 | .943 | .849 |
|  | 29.9450 | .937 | .849 |
|  | 29.9650 | .931 | .843 |
|  | 30.0100 | .931 | .836 |
|  | 30.0650 | .925 | .836 |
|  | 30.1200 | .918 | .836 |
|  | 30.1700 | .918 | .830 |
|  | 30.2300 | .918 | .824 |
|  | 30.2900 | .912 | .824 |
|  | 30.3150 | .906 | .824 |
|  | 30.3400 | .906 | .818 |
|  | 30.3650 | .906 | .811 |
|  | 30.3800 | .906 | .805 |
|  | 30.4200 | .906 | .799 |
|  | 30.4550 | .906 | .792 |
|  | 30.5250 | .906 | .786 |
|  | 30.6150 | .906 | .780 |
|  | 30.6550 | .906 | .774 |
|  | 30.6750 | .899 | .774 |
|  | 30.7100 | .899 | .767 |
|  | 30.7650 | .899 | .761 |
|  | 30.7950 | .899 | .755 |
|  | 30.8050 | .893 | .755 |
|  | 30.8300 | .887 | .755 |
|  | 30.8650 | .887 | .742 |
|  | 30.9500 | .881 | .742 |
|  | 31.0450 | .881 | .736 |
|  | 31.0800 | .881 | .730 |
|  | 31.1100 | .881 | .723 |
|  | 31.1350 | .881 | .717 |
|  | 31.1450 | .881 | .711 |
|  | 31.1650 | .881 | .704 |
|  | 31.1900 | .881 | .698 |
|  | 31.2100 | .881 | .692 |
|  | 31.2450 | .881 | .686 |
|  | 31.2800 | .874 | .686 |
|  | 31.3100 | .868 | .686 |
|  | 31.3650 | .868 | .679 |
|  | 31.4200 | .868 | .673 |
|  | 31.4750 | .862 | .673 |
|  | 31.5200 | .862 | .667 |
|  | 31.5400 | .855 | .667 |
|  | 31.5550 | .855 | .660 |
|  | 31.5800 | .855 | .654 |
|  | 31.6050 | .855 | .648 |
|  | 31.6150 | .855 | .642 |
|  | 31.6350 | .855 | .635 |
|  | 31.6600 | .849 | .635 |
|  | 31.6950 | .849 | .616 |
|  | 31.7250 | .843 | .610 |
|  | 31.7350 | .843 | .604 |
|  | 31.7450 | .843 | .597 |
|  | 31.7550 | .843 | .591 |
|  | 31.7850 | .843 | .585 |
|  | 31.8400 | .836 | .585 |
|  | 31.8750 | .830 | .585 |
|  | 31.8950 | .830 | .579 |
|  | 31.9150 | .830 | .572 |
|  | 31.9300 | .830 | .566 |
|  | 31.9600 | .830 | .560 |
|  | 32.0400 | .830 | .553 |
|  | 32.1150 | .830 | .547 |
|  | 32.1350 | .824 | .547 |
|  | 32.1700 | .824 | .541 |
|  | 32.2200 | .824 | .535 |
|  | 32.2700 | .824 | .528 |
|  | 32.3100 | .818 | .516 |
|  | 32.3300 | .811 | .516 |
|  | 32.3450 | .805 | .516 |
|  | 32.3750 | .799 | .509 |
|  | 32.4250 | .799 | .503 |
|  | 32.4550 | .799 | .497 |
|  | 32.4650 | .786 | .497 |
|  | 32.4750 | .786 | .491 |
|  | 32.4850 | .780 | .491 |
|  | 32.5050 | .780 | .478 |
|  | 32.5400 | .780 | .472 |
|  | 32.5650 | .767 | .472 |
|  | 32.5850 | .767 | .465 |
|  | 32.6050 | .755 | .465 |
|  | 32.6150 | .755 | .459 |
|  | 32.6300 | .755 | .453 |
|  | 32.6450 | .755 | .447 |
|  | 32.6850 | .755 | .440 |
|  | 32.7250 | .755 | .434 |
|  | 32.7350 | .755 | .428 |
|  | 32.7450 | .748 | .428 |
|  | 32.7650 | .742 | .428 |
|  | 32.7850 | .742 | .421 |
|  | 32.8000 | .742 | .415 |
|  | 32.8350 | .736 | .415 |
|  | 32.8900 | .730 | .415 |
|  | 32.9400 | .723 | .415 |
|  | 32.9650 | .723 | .409 |
|  | 32.9800 | .717 | .409 |
|  | 33.0250 | .711 | .409 |
|  | 33.0750 | .711 | .403 |
|  | 33.1250 | .704 | .403 |
|  | 33.1700 | .704 | .396 |
|  | 33.1900 | .704 | .390 |
|  | 33.2100 | .704 | .384 |
|  | 33.2250 | .698 | .377 |
|  | 33.2400 | .698 | .371 |
|  | 33.2550 | .698 | .365 |
|  | 33.2700 | .698 | .358 |
|  | 33.2850 | .692 | .352 |
|  | 33.3050 | .692 | .340 |
|  | 33.3250 | .692 | .333 |
|  | 33.3450 | .686 | .321 |
|  | 33.4150 | .679 | .321 |
|  | 33.4800 | .679 | .314 |
|  | 33.5000 | .673 | .314 |
|  | 33.5150 | .667 | .314 |
|  | 33.5250 | .660 | .314 |
|  | 33.5700 | .660 | .308 |
|  | 33.6200 | .660 | .302 |
|  | 33.6350 | .660 | .289 |
|  | 33.6450 | .660 | .283 |
|  | 33.6550 | .654 | .277 |
|  | 33.6800 | .654 | .270 |
|  | 33.7050 | .648 | .270 |
|  | 33.7250 | .642 | .264 |
|  | 33.7500 | .642 | .252 |
|  | 33.7750 | .642 | .245 |
|  | 33.8050 | .629 | .245 |
|  | 33.8500 | .623 | .239 |
|  | 33.9250 | .623 | .233 |
|  | 33.9750 | .623 | .226 |
|  | 34.0050 | .623 | .220 |
|  | 34.0550 | .623 | .208 |
|  | 34.0950 | .616 | .208 |
|  | 34.1150 | .616 | .201 |
|  | 34.1550 | .616 | .189 |
|  | 34.2050 | .610 | .189 |
|  | 34.2500 | .610 | .176 |
|  | 34.2850 | .610 | .170 |
|  | 34.3050 | .591 | .170 |
|  | 34.3350 | .591 | .164 |
|  | 34.3800 | .591 | .157 |
|  | 34.4250 | .585 | .157 |
|  | 34.4850 | .579 | .157 |
|  | 34.6200 | .572 | .157 |
|  | 34.7200 | .572 | .151 |
|  | 34.7600 | .572 | .138 |
|  | 34.8300 | .572 | .132 |
|  | 34.9300 | .566 | .132 |
|  | 35.0050 | .566 | .126 |
|  | 35.0250 | .566 | .119 |
|  | 35.0650 | .566 | .113 |
|  | 35.1050 | .560 | .107 |
|  | 35.1850 | .553 | .107 |
|  | 35.3050 | .547 | .107 |
|  | 35.3550 | .535 | .107 |
|  | 35.4000 | .528 | .107 |
|  | 35.4500 | .522 | .107 |
|  | 35.4700 | .522 | .101 |
|  | 35.4950 | .516 | .101 |
|  | 35.5300 | .509 | .101 |
|  | 35.5850 | .503 | .101 |
|  | 35.6250 | .503 | .094 |
|  | 35.6400 | .497 | .094 |
|  | 35.6600 | .497 | .088 |
|  | 35.6800 | .497 | .082 |
|  | 35.7150 | .497 | .069 |
|  | 35.7450 | .491 | .069 |
|  | 35.7950 | .484 | .069 |
|  | 35.8750 | .478 | .069 |
|  | 35.9150 | .472 | .069 |
|  | 35.9750 | .465 | .069 |
|  | 36.0800 | .465 | .063 |
|  | 36.1350 | .465 | .057 |
|  | 36.1650 | .459 | .050 |
|  | 36.2300 | .459 | .044 |
|  | 36.3000 | .453 | .044 |
|  | 36.3350 | .453 | .038 |
|  | 36.3600 | .453 | .031 |
|  | 36.4050 | .447 | .031 |
|  | 36.4400 | .440 | .031 |
|  | 36.4600 | .428 | .031 |
|  | 36.4750 | .428 | .025 |
|  | 36.4850 | .428 | .019 |
|  | 36.5050 | .421 | .019 |
|  | 36.5650 | .415 | .019 |
|  | 36.6150 | .409 | .019 |
|  | 36.6550 | .403 | .019 |
|  | 36.7500 | .396 | .019 |
|  | 36.8150 | .390 | .019 |
|  | 36.9000 | .384 | .019 |
|  | 37.0000 | .384 | .013 |
|  | 37.0600 | .377 | .013 |
|  | 37.1200 | .371 | .013 |
|  | 37.1950 | .371 | .006 |
|  | 37.2550 | .365 | .006 |
|  | 37.2950 | .358 | .006 |
|  | 37.3950 | .352 | .006 |
|  | 37.6450 | .346 | .006 |
|  | 37.8550 | .340 | .006 |
|  | 37.9400 | .333 | .006 |
|  | 38.0150 | .333 | .000 |
|  | 38.0350 | .327 | .000 |
|  | 38.0700 | .321 | .000 |
|  | 38.1900 | .314 | .000 |
|  | 38.3800 | .308 | .000 |
|  | 38.5050 | .302 | .000 |
|  | 38.5550 | .296 | .000 |
|  | 38.5900 | .289 | .000 |
|  | 38.6700 | .283 | .000 |
|  | 38.7500 | .277 | .000 |
|  | 38.7850 | .270 | .000 |
|  | 38.9050 | .264 | .000 |
|  | 39.0100 | .258 | .000 |
|  | 39.0300 | .252 | .000 |
|  | 39.0600 | .245 | .000 |
|  | 39.1050 | .239 | .000 |
|  | 39.1750 | .233 | .000 |
|  | 39.2450 | .226 | .000 |
|  | 39.2750 | .220 | .000 |
|  | 39.4100 | .214 | .000 |
|  | 39.5450 | .208 | .000 |
|  | 39.5700 | .201 | .000 |
|  | 39.6350 | .195 | .000 |
|  | 39.7550 | .182 | .000 |
|  | 39.8400 | .170 | .000 |
|  | 39.8850 | .164 | .000 |
|  | 39.9550 | .151 | .000 |
|  | 40.0000 | .145 | .000 |
|  | 40.1300 | .138 | .000 |
|  | 40.4450 | .132 | .000 |
|  | 40.6600 | .126 | .000 |
|  | 40.6850 | .119 | .000 |
|  | 40.7700 | .113 | .000 |
|  | 40.9050 | .107 | .000 |
|  | 41.0200 | .101 | .000 |
|  | 41.1050 | .094 | .000 |
|  | 41.1700 | .088 | .000 |
|  | 41.4200 | .082 | .000 |
|  | 41.9800 | .075 | .000 |
|  | 42.3350 | .069 | .000 |
|  | 42.5000 | .057 | .000 |
|  | 42.6750 | .050 | .000 |
|  | 42.7800 | .044 | .000 |
|  | 43.0850 | .038 | .000 |
|  | 43.7450 | .031 | .000 |
|  | 44.5250 | .025 | .000 |
|  | 45.0700 | .019 | .000 |
|  | 45.6550 | .013 | .000 |
|  | 46.7650 | .006 | .000 |
|  | 48.5000 | .000 | .000 |
| Width Left | 16.0400 | 1.000 | 1.000 |
|  | 17.4650 | 1.000 | .994 |
|  | 18.4650 | 1.000 | .987 |
|  | 19.1600 | 1.000 | .981 |
|  | 19.4900 | 1.000 | .975 |
|  | 19.9750 | 1.000 | .969 |
|  | 20.3150 | 1.000 | .962 |
|  | 20.4400 | 1.000 | .956 |
|  | 20.9450 | 1.000 | .950 |
|  | 21.4850 | 1.000 | .943 |
|  | 21.7650 | 1.000 | .937 |
|  | 22.0800 | 1.000 | .931 |
|  | 22.2500 | 1.000 | .925 |
|  | 22.3350 | 1.000 | .918 |
|  | 22.4450 | 1.000 | .912 |
|  | 22.5250 | 1.000 | .906 |
|  | 22.5800 | 1.000 | .899 |
|  | 22.6250 | 1.000 | .893 |
|  | 22.6500 | 1.000 | .887 |
|  | 22.7050 | 1.000 | .881 |
|  | 22.8500 | 1.000 | .874 |
|  | 22.9700 | 1.000 | .868 |
|  | 23.0050 | 1.000 | .855 |
|  | 23.3950 | 1.000 | .849 |
|  | 23.8000 | 1.000 | .843 |
|  | 23.8700 | 1.000 | .836 |
|  | 23.9950 | 1.000 | .830 |
|  | 24.1350 | 1.000 | .824 |
|  | 24.1950 | 1.000 | .818 |
|  | 24.2850 | 1.000 | .811 |
|  | 24.3650 | 1.000 | .805 |
|  | 24.3900 | .994 | .805 |
|  | 24.4350 | .987 | .805 |
|  | 24.4950 | .987 | .799 |
|  | 24.5550 | .987 | .792 |
|  | 24.6900 | .987 | .786 |
|  | 24.8350 | .987 | .780 |
|  | 24.8950 | .987 | .774 |
|  | 24.9350 | .987 | .767 |
|  | 24.9550 | .987 | .761 |
|  | 24.9900 | .987 | .755 |
|  | 25.1150 | .987 | .748 |
|  | 25.2500 | .987 | .742 |
|  | 25.3250 | .987 | .736 |
|  | 25.4300 | .987 | .730 |
|  | 25.5300 | .987 | .723 |
|  | 25.6400 | .987 | .717 |
|  | 25.7400 | .987 | .711 |
|  | 25.7950 | .987 | .704 |
|  | 25.8450 | .987 | .698 |
|  | 25.8850 | .987 | .692 |
|  | 25.9800 | .987 | .686 |
|  | 26.0750 | .987 | .679 |
|  | 26.1450 | .987 | .667 |
|  | 26.2000 | .987 | .660 |
|  | 26.2250 | .987 | .654 |
|  | 26.2450 | .987 | .648 |
|  | 26.3200 | .987 | .642 |
|  | 26.4100 | .987 | .635 |
|  | 26.4700 | .987 | .629 |
|  | 26.5400 | .987 | .623 |
|  | 26.5750 | .987 | .610 |
|  | 26.5950 | .987 | .604 |
|  | 26.6600 | .987 | .597 |
|  | 26.7450 | .987 | .585 |
|  | 26.8050 | .987 | .579 |
|  | 26.8700 | .987 | .572 |
|  | 26.9250 | .987 | .566 |
|  | 26.9600 | .987 | .560 |
|  | 26.9850 | .987 | .553 |
|  | 27.1650 | .987 | .547 |
|  | 27.3500 | .987 | .535 |
|  | 27.4200 | .987 | .528 |
|  | 27.5250 | .987 | .522 |
|  | 27.5750 | .975 | .522 |
|  | 27.5850 | .975 | .516 |
|  | 27.6500 | .975 | .509 |
|  | 27.7400 | .975 | .503 |
|  | 27.8850 | .975 | .497 |
|  | 28.0400 | .969 | .497 |
|  | 28.0850 | .969 | .491 |
|  | 28.1100 | .969 | .484 |
|  | 28.1950 | .962 | .484 |
|  | 28.3050 | .956 | .484 |
|  | 28.3550 | .956 | .478 |
|  | 28.3750 | .956 | .472 |
|  | 28.4650 | .956 | .465 |
|  | 28.5450 | .950 | .465 |
|  | 28.5550 | .950 | .459 |
|  | 28.5700 | .943 | .459 |
|  | 28.5850 | .937 | .459 |
|  | 28.6200 | .931 | .459 |
|  | 28.6900 | .931 | .453 |
|  | 28.7450 | .925 | .447 |
|  | 28.7650 | .925 | .440 |
|  | 28.7950 | .925 | .434 |
|  | 28.8250 | .925 | .428 |
|  | 28.8650 | .925 | .421 |
|  | 28.9150 | .925 | .415 |
|  | 28.9800 | .925 | .409 |
|  | 29.0450 | .918 | .409 |
|  | 29.0700 | .918 | .403 |
|  | 29.1050 | .912 | .403 |
|  | 29.1400 | .906 | .403 |
|  | 29.2100 | .906 | .396 |
|  | 29.2800 | .906 | .390 |
|  | 29.3000 | .906 | .384 |
|  | 29.3650 | .906 | .377 |
|  | 29.4300 | .899 | .371 |
|  | 29.4550 | .893 | .371 |
|  | 29.5000 | .893 | .365 |
|  | 29.5400 | .887 | .365 |
|  | 29.5550 | .887 | .358 |
|  | 29.5700 | .868 | .358 |
|  | 29.6100 | .862 | .358 |
|  | 29.6700 | .862 | .352 |
|  | 29.7050 | .855 | .352 |
|  | 29.7300 | .855 | .346 |
|  | 29.7550 | .843 | .346 |
|  | 29.7850 | .843 | .340 |
|  | 29.8200 | .836 | .340 |
|  | 29.8550 | .836 | .333 |
|  | 29.9050 | .830 | .327 |
|  | 29.9400 | .830 | .321 |
|  | 29.9550 | .824 | .321 |
|  | 29.9700 | .818 | .321 |
|  | 29.9850 | .805 | .321 |
|  | 30.0050 | .805 | .314 |
|  | 30.0250 | .799 | .314 |
|  | 30.0550 | .799 | .308 |
|  | 30.0900 | .799 | .302 |
|  | 30.1100 | .799 | .296 |
|  | 30.1500 | .799 | .289 |
|  | 30.1850 | .792 | .289 |
|  | 30.2250 | .792 | .283 |
|  | 30.2650 | .792 | .277 |
|  | 30.2850 | .792 | .270 |
|  | 30.3100 | .786 | .264 |
|  | 30.3250 | .780 | .264 |
|  | 30.3350 | .780 | .258 |
|  | 30.3900 | .774 | .258 |
|  | 30.4700 | .767 | .252 |
|  | 30.5100 | .761 | .245 |
|  | 30.5350 | .755 | .233 |
|  | 30.5550 | .755 | .226 |
|  | 30.5700 | .755 | .220 |
|  | 30.5900 | .748 | .220 |
|  | 30.6050 | .748 | .214 |
|  | 30.6450 | .742 | .214 |
|  | 30.7100 | .742 | .208 |
|  | 30.7450 | .742 | .201 |
|  | 30.8050 | .742 | .195 |
|  | 30.8950 | .736 | .195 |
|  | 30.9550 | .723 | .195 |
|  | 30.9900 | .723 | .189 |
|  | 31.0200 | .723 | .182 |
|  | 31.0500 | .723 | .176 |
|  | 31.0850 | .717 | .176 |
|  | 31.1500 | .711 | .176 |
|  | 31.2050 | .698 | .176 |
|  | 31.2300 | .698 | .170 |
|  | 31.2450 | .698 | .164 |
|  | 31.2550 | .686 | .164 |
|  | 31.2750 | .679 | .164 |
|  | 31.3350 | .673 | .157 |
|  | 31.3900 | .667 | .157 |
|  | 31.4200 | .654 | .157 |
|  | 31.4550 | .648 | .157 |
|  | 31.4750 | .648 | .151 |
|  | 31.5100 | .642 | .151 |
|  | 31.5500 | .635 | .151 |
|  | 31.5650 | .629 | .151 |
|  | 31.6200 | .629 | .145 |
|  | 31.6850 | .629 | .138 |
|  | 31.7100 | .629 | .132 |
|  | 31.7450 | .623 | .132 |
|  | 31.8100 | .616 | .132 |
|  | 31.8550 | .610 | .132 |
|  | 31.8650 | .610 | .119 |
|  | 31.8800 | .604 | .119 |
|  | 31.8950 | .597 | .119 |
|  | 31.9150 | .591 | .119 |
|  | 31.9350 | .585 | .119 |
|  | 31.9550 | .579 | .119 |
|  | 31.9800 | .572 | .119 |
|  | 32.0000 | .572 | .113 |
|  | 32.0300 | .566 | .113 |
|  | 32.0750 | .560 | .113 |
|  | 32.1100 | .553 | .113 |
|  | 32.1250 | .547 | .113 |
|  | 32.1350 | .541 | .113 |
|  | 32.1450 | .535 | .113 |
|  | 32.1600 | .528 | .113 |
|  | 32.1750 | .522 | .113 |
|  | 32.1900 | .522 | .107 |
|  | 32.2100 | .516 | .107 |
|  | 32.2350 | .497 | .107 |
|  | 32.2650 | .491 | .107 |
|  | 32.3000 | .484 | .107 |
|  | 32.3250 | .478 | .107 |
|  | 32.3450 | .472 | .107 |
|  | 32.3650 | .465 | .107 |
|  | 32.3750 | .459 | .107 |
|  | 32.3950 | .453 | .107 |
|  | 32.4150 | .447 | .107 |
|  | 32.4450 | .447 | .101 |
|  | 32.4800 | .440 | .101 |
|  | 32.5000 | .434 | .101 |
|  | 32.5150 | .428 | .101 |
|  | 32.5250 | .421 | .101 |
|  | 32.5400 | .415 | .101 |
|  | 32.5700 | .409 | .101 |
|  | 32.6000 | .403 | .101 |
|  | 32.6400 | .396 | .101 |
|  | 32.6900 | .390 | .101 |
|  | 32.7650 | .384 | .101 |
|  | 32.8600 | .377 | .101 |
|  | 32.9450 | .358 | .101 |
|  | 33.0300 | .352 | .101 |
|  | 33.1050 | .346 | .094 |
|  | 33.1500 | .340 | .094 |
|  | 33.1700 | .333 | .094 |
|  | 33.1850 | .327 | .094 |
|  | 33.1950 | .321 | .094 |
|  | 33.2050 | .321 | .088 |
|  | 33.2150 | .314 | .088 |
|  | 33.2500 | .296 | .088 |
|  | 33.2950 | .289 | .088 |
|  | 33.3300 | .283 | .088 |
|  | 33.4500 | .277 | .088 |
|  | 33.5650 | .270 | .088 |
|  | 33.5850 | .264 | .088 |
|  | 33.6050 | .258 | .088 |
|  | 33.6500 | .252 | .088 |
|  | 33.7150 | .245 | .088 |
|  | 33.7600 | .239 | .082 |
|  | 33.8000 | .233 | .082 |
|  | 33.8400 | .226 | .082 |
|  | 33.8550 | .220 | .082 |
|  | 33.8650 | .214 | .082 |
|  | 33.9650 | .208 | .082 |
|  | 34.0750 | .201 | .082 |
|  | 34.1200 | .195 | .075 |
|  | 34.2000 | .189 | .075 |
|  | 34.2550 | .176 | .069 |
|  | 34.2750 | .170 | .069 |
|  | 34.2950 | .170 | .063 |
|  | 34.3250 | .164 | .063 |
|  | 34.3550 | .157 | .063 |
|  | 34.4000 | .151 | .063 |
|  | 34.4450 | .145 | .063 |
|  | 34.4550 | .138 | .063 |
|  | 34.4850 | .132 | .063 |
|  | 34.5400 | .126 | .063 |
|  | 34.5850 | .126 | .057 |
|  | 34.6100 | .119 | .057 |
|  | 34.7100 | .113 | .057 |
|  | 34.8800 | .107 | .057 |
|  | 35.0100 | .101 | .057 |
|  | 35.0750 | .094 | .057 |
|  | 35.0950 | .088 | .057 |
|  | 35.1250 | .082 | .057 |
|  | 35.1550 | .075 | .057 |
|  | 35.1950 | .069 | .057 |
|  | 35.2550 | .069 | .050 |
|  | 35.3050 | .063 | .050 |
|  | 35.3600 | .057 | .050 |
|  | 35.4200 | .057 | .044 |
|  | 35.4600 | .050 | .044 |
|  | 35.5000 | .050 | .038 |
|  | 35.5700 | .044 | .038 |
|  | 35.6600 | .044 | .031 |
|  | 35.7500 | .038 | .031 |
|  | 35.7950 | .038 | .025 |
|  | 35.8550 | .031 | .025 |
|  | 35.9150 | .031 | .019 |
|  | 35.9350 | .025 | .019 |
|  | 35.9600 | .025 | .013 |
|  | 35.9800 | .019 | .013 |
|  | 36.3250 | .013 | .013 |
|  | 37.0600 | .006 | .013 |
|  | 37.6150 | .006 | .006 |
|  | 40.1500 | .000 | .006 |
|  | 43.5300 | .000 | .000 |
| Volume Left | 6394.0100 | 1.000 | 1.000 |
|  | 7003.5900 | 1.000 | .994 |
|  | 7666.3950 | 1.000 | .987 |
|  | 7890.0300 | 1.000 | .981 |
|  | 8148.4750 | 1.000 | .975 |
|  | 8265.0450 | 1.000 | .969 |
|  | 8467.3650 | 1.000 | .962 |
|  | 8694.1850 | .994 | .962 |
|  | 8960.7400 | .987 | .962 |
|  | 9200.5300 | .987 | .956 |
|  | 9242.3800 | .981 | .956 |
|  | 9261.7750 | .981 | .950 |
|  | 9279.8750 | .981 | .943 |
|  | 9300.0550 | .981 | .937 |
|  | 9313.3700 | .975 | .937 |
|  | 9370.2950 | .975 | .931 |
|  | 9489.6700 | .975 | .925 |
|  | 9619.8850 | .975 | .918 |
|  | 9700.8700 | .975 | .912 |
|  | 9778.5400 | .975 | .906 |
|  | 9857.3500 | .975 | .899 |
|  | 9899.2450 | .975 | .893 |
|  | 9934.5750 | .975 | .887 |
|  | 10020.6350 | .975 | .881 |
|  | 10093.4100 | .975 | .874 |
|  | 10108.1800 | .975 | .868 |
|  | 10121.4200 | .975 | .862 |
|  | 10126.6350 | .975 | .855 |
|  | 10140.3400 | .975 | .849 |
|  | 10182.5050 | .975 | .843 |
|  | 10216.3800 | .975 | .836 |
|  | 10261.6450 | .975 | .830 |
|  | 10321.1050 | .975 | .824 |
|  | 10399.6500 | .975 | .818 |
|  | 10481.8250 | .969 | .818 |
|  | 10566.9300 | .969 | .811 |
|  | 10651.2200 | .969 | .805 |
|  | 10672.9600 | .969 | .799 |
|  | 10680.5800 | .969 | .792 |
|  | 10732.5250 | .969 | .786 |
|  | 10807.6550 | .969 | .780 |
|  | 10844.9050 | .962 | .780 |
|  | 10855.0600 | .962 | .774 |
|  | 10861.1750 | .962 | .767 |
|  | 10872.3400 | .962 | .761 |
|  | 10909.3250 | .962 | .755 |
|  | 10954.9850 | .962 | .748 |
|  | 10987.1000 | .962 | .742 |
|  | 11012.8800 | .962 | .736 |
|  | 11023.1250 | .962 | .730 |
|  | 11029.2700 | .956 | .730 |
|  | 11051.3200 | .956 | .723 |
|  | 11072.8650 | .956 | .717 |
|  | 11080.5400 | .956 | .711 |
|  | 11090.9500 | .950 | .711 |
|  | 11129.3200 | .950 | .704 |
|  | 11182.6000 | .950 | .698 |
|  | 11239.5650 | .943 | .698 |
|  | 11330.4050 | .943 | .692 |
|  | 11393.3700 | .943 | .686 |
|  | 11419.7200 | .937 | .686 |
|  | 11438.2700 | .937 | .679 |
|  | 11444.3750 | .937 | .673 |
|  | 11500.4600 | .931 | .673 |
|  | 11570.0000 | .931 | .667 |
|  | 11603.2500 | .931 | .660 |
|  | 11629.2350 | .931 | .654 |
|  | 11647.1950 | .931 | .648 |
|  | 11663.4950 | .931 | .642 |
|  | 11685.6950 | .925 | .642 |
|  | 11716.6300 | .925 | .635 |
|  | 11766.6450 | .925 | .629 |
|  | 11809.4700 | .925 | .623 |
|  | 11832.7250 | .925 | .616 |
|  | 11864.3750 | .925 | .610 |
|  | 11900.6400 | .925 | .604 |
|  | 11917.6650 | .925 | .597 |
|  | 11923.6950 | .925 | .591 |
|  | 11943.3050 | .918 | .591 |
|  | 11959.4400 | .918 | .585 |
|  | 11962.3850 | .918 | .579 |
|  | 11965.4150 | .912 | .579 |
|  | 11968.3500 | .912 | .572 |
|  | 11988.4350 | .912 | .566 |
|  | 12008.5600 | .906 | .566 |
|  | 12040.3400 | .906 | .560 |
|  | 12113.7050 | .899 | .560 |
|  | 12176.8450 | .899 | .553 |
|  | 12200.4350 | .893 | .553 |
|  | 12227.5050 | .893 | .547 |
|  | 12262.0400 | .887 | .547 |
|  | 12307.7850 | .887 | .541 |
|  | 12351.9750 | .887 | .535 |
|  | 12373.2000 | .881 | .535 |
|  | 12386.0700 | .874 | .535 |
|  | 12390.4000 | .874 | .528 |
|  | 12404.1100 | .868 | .528 |
|  | 12421.5000 | .868 | .522 |
|  | 12437.4200 | .868 | .516 |
|  | 12447.3950 | .862 | .516 |
|  | 12452.7300 | .855 | .516 |
|  | 12458.6650 | .855 | .509 |
|  | 12476.7150 | .855 | .503 |
|  | 12500.2450 | .855 | .497 |
|  | 12518.4250 | .849 | .497 |
|  | 12530.9500 | .849 | .491 |
|  | 12562.8050 | .843 | .491 |
|  | 12597.8850 | .843 | .484 |
|  | 12621.6150 | .836 | .484 |
|  | 12642.8350 | .830 | .484 |
|  | 12684.7500 | .824 | .484 |
|  | 12726.5400 | .818 | .484 |
|  | 12738.5750 | .818 | .478 |
|  | 12749.4550 | .818 | .472 |
|  | 12762.3550 | .811 | .472 |
|  | 12782.8500 | .811 | .465 |
|  | 12827.5450 | .805 | .465 |
|  | 12874.3300 | .799 | .465 |
|  | 12888.5250 | .792 | .465 |
|  | 12894.6400 | .792 | .459 |
|  | 12900.7100 | .792 | .453 |
|  | 12911.0650 | .792 | .447 |
|  | 12938.9500 | .786 | .447 |
|  | 12981.0750 | .780 | .447 |
|  | 13013.7100 | .780 | .440 |
|  | 13042.6350 | .774 | .440 |
|  | 13063.6500 | .767 | .440 |
|  | 13087.6850 | .761 | .440 |
|  | 13108.8050 | .755 | .440 |
|  | 13117.3250 | .755 | .434 |
|  | 13141.9600 | .748 | .434 |
|  | 13219.6050 | .742 | .434 |
|  | 13287.0950 | .742 | .428 |
|  | 13304.2800 | .742 | .421 |
|  | 13325.8250 | .736 | .421 |
|  | 13342.2750 | .736 | .415 |
|  | 13353.2350 | .730 | .415 |
|  | 13392.0900 | .723 | .415 |
|  | 13435.0950 | .717 | .415 |
|  | 13457.5100 | .717 | .409 |
|  | 13481.8500 | .717 | .403 |
|  | 13501.9250 | .717 | .396 |
|  | 13523.1900 | .711 | .396 |
|  | 13561.0700 | .711 | .390 |
|  | 13625.0900 | .704 | .390 |
|  | 13689.9900 | .704 | .384 |
|  | 13718.3700 | .698 | .384 |
|  | 13722.5800 | .692 | .384 |
|  | 13741.1050 | .686 | .384 |
|  | 13761.0500 | .686 | .377 |
|  | 13764.2700 | .686 | .371 |
|  | 13772.3350 | .686 | .365 |
|  | 13813.1800 | .679 | .365 |
|  | 13867.8950 | .679 | .358 |
|  | 13914.1700 | .679 | .352 |
|  | 13957.7000 | .673 | .352 |
|  | 13989.3550 | .667 | .352 |
|  | 14002.8900 | .667 | .346 |
|  | 14008.2150 | .667 | .340 |
|  | 14017.3000 | .667 | .333 |
|  | 14048.1300 | .667 | .327 |
|  | 14084.0700 | .660 | .327 |
|  | 14130.1050 | .660 | .321 |
|  | 14195.2650 | .660 | .314 |
|  | 14225.0300 | .660 | .308 |
|  | 14243.7800 | .654 | .308 |
|  | 14281.0500 | .654 | .302 |
|  | 14316.1350 | .654 | .296 |
|  | 14356.0100 | .648 | .296 |
|  | 14394.6350 | .648 | .289 |
|  | 14414.4800 | .642 | .289 |
|  | 14441.0950 | .642 | .283 |
|  | 14515.3650 | .635 | .283 |
|  | 14578.1000 | .629 | .283 |
|  | 14588.4850 | .623 | .283 |
|  | 14591.6200 | .616 | .283 |
|  | 14620.2150 | .616 | .277 |
|  | 14646.7550 | .610 | .277 |
|  | 14652.8800 | .604 | .277 |
|  | 14676.5800 | .604 | .270 |
|  | 14697.1150 | .597 | .270 |
|  | 14723.0750 | .597 | .264 |
|  | 14756.5450 | .591 | .264 |
|  | 14777.8600 | .585 | .264 |
|  | 14794.1750 | .585 | .258 |
|  | 14806.0400 | .579 | .258 |
|  | 14820.2300 | .572 | .258 |
|  | 14827.5350 | .566 | .258 |
|  | 14833.9250 | .560 | .258 |
|  | 14843.3800 | .553 | .258 |
|  | 14846.9100 | .553 | .252 |
|  | 14864.3050 | .547 | .252 |
|  | 14901.3800 | .541 | .252 |
|  | 14931.6500 | .535 | .252 |
|  | 14949.5350 | .528 | .252 |
|  | 14960.2900 | .522 | .252 |
|  | 14977.7700 | .522 | .245 |
|  | 14995.4550 | .522 | .239 |
|  | 15008.0750 | .522 | .233 |
|  | 15046.7450 | .516 | .233 |
|  | 15077.0200 | .516 | .226 |
|  | 15099.3500 | .509 | .226 |
|  | 15150.3600 | .509 | .220 |
|  | 15189.8850 | .503 | .220 |
|  | 15201.5750 | .503 | .214 |
|  | 15206.1650 | .503 | .208 |
|  | 15218.3750 | .503 | .201 |
|  | 15231.8300 | .497 | .201 |
|  | 15246.5150 | .491 | .201 |
|  | 15257.9100 | .484 | .201 |
|  | 15260.6100 | .478 | .201 |
|  | 15331.8700 | .478 | .195 |
|  | 15408.2200 | .478 | .189 |
|  | 15417.3750 | .472 | .189 |
|  | 15440.7500 | .465 | .189 |
|  | 15467.8250 | .459 | .189 |
|  | 15487.6550 | .453 | .189 |
|  | 15512.3250 | .447 | .189 |
|  | 15531.5400 | .447 | .182 |
|  | 15543.7500 | .440 | .182 |
|  | 15582.9900 | .434 | .182 |
|  | 15629.8000 | .428 | .182 |
|  | 15662.3200 | .428 | .176 |
|  | 15698.3050 | .421 | .176 |
|  | 15724.2400 | .415 | .176 |
|  | 15738.7100 | .409 | .176 |
|  | 15746.0500 | .403 | .176 |
|  | 15759.7250 | .396 | .176 |
|  | 15774.0550 | .390 | .176 |
|  | 15783.7550 | .390 | .170 |
|  | 15796.8300 | .390 | .164 |
|  | 15815.3000 | .384 | .164 |
|  | 15837.2300 | .384 | .157 |
|  | 15849.3250 | .384 | .151 |
|  | 15919.4050 | .377 | .151 |
|  | 16032.6300 | .377 | .145 |
|  | 16091.5200 | .377 | .138 |
|  | 16125.7550 | .371 | .138 |
|  | 16146.5600 | .365 | .138 |
|  | 16234.4200 | .365 | .132 |
|  | 16339.4700 | .358 | .132 |
|  | 16389.5500 | .352 | .132 |
|  | 16426.2850 | .346 | .132 |
|  | 16432.8700 | .340 | .132 |
|  | 16439.2950 | .340 | .126 |
|  | 16476.2350 | .333 | .126 |
|  | 16513.4450 | .333 | .119 |
|  | 16524.8100 | .327 | .119 |
|  | 16546.7850 | .321 | .119 |
|  | 16567.8150 | .314 | .119 |
|  | 16587.7100 | .308 | .119 |
|  | 16608.7100 | .302 | .119 |
|  | 16615.9100 | .296 | .119 |
|  | 16619.2350 | .289 | .119 |
|  | 16632.3700 | .289 | .113 |
|  | 16700.6600 | .283 | .113 |
|  | 16759.1900 | .277 | .113 |
|  | 16788.4950 | .270 | .113 |
|  | 16831.3550 | .264 | .113 |
|  | 16849.0650 | .258 | .113 |
|  | 16859.6250 | .252 | .113 |
|  | 16876.2500 | .252 | .107 |
|  | 16892.9600 | .245 | .107 |
|  | 16919.8150 | .239 | .107 |
|  | 16952.7050 | .239 | .101 |
|  | 16967.5550 | .239 | .094 |
|  | 17029.1100 | .233 | .094 |
|  | 17096.2050 | .226 | .094 |
|  | 17118.8850 | .226 | .088 |
|  | 17136.0950 | .220 | .088 |
|  | 17141.6700 | .214 | .088 |
|  | 17161.7000 | .208 | .088 |
|  | 17194.2750 | .201 | .088 |
|  | 17262.0950 | .195 | .088 |
|  | 17328.3000 | .189 | .088 |
|  | 17345.3550 | .182 | .088 |
|  | 17366.1000 | .176 | .088 |
|  | 17391.1550 | .176 | .082 |
|  | 17402.9350 | .170 | .082 |
|  | 17416.6500 | .164 | .082 |
|  | 17443.5850 | .157 | .082 |
|  | 17467.3050 | .151 | .082 |
|  | 17498.7450 | .151 | .075 |
|  | 17556.4750 | .151 | .069 |
|  | 17595.7800 | .145 | .069 |
|  | 17613.4200 | .138 | .069 |
|  | 17636.7300 | .132 | .069 |
|  | 17659.5150 | .126 | .069 |
|  | 17712.5450 | .126 | .063 |
|  | 17761.8500 | .119 | .063 |
|  | 17792.0300 | .119 | .057 |
|  | 17831.5950 | .119 | .050 |
|  | 17896.5500 | .119 | .044 |
|  | 18005.7600 | .119 | .038 |
|  | 18109.3600 | .113 | .038 |
|  | 18166.1500 | .113 | .031 |
|  | 18184.3100 | .107 | .031 |
|  | 18204.1950 | .101 | .031 |
|  | 18239.8350 | .094 | .031 |
|  | 18307.0950 | .088 | .031 |
|  | 18405.0400 | .082 | .031 |
|  | 18468.2800 | .075 | .031 |
|  | 18536.2850 | .069 | .031 |
|  | 18660.4250 | .063 | .031 |
|  | 18764.5200 | .057 | .031 |
|  | 18852.2150 | .050 | .031 |
|  | 18923.4000 | .050 | .025 |
|  | 18957.9050 | .044 | .025 |
|  | 18987.0550 | .044 | .019 |
|  | 19066.6050 | .044 | .013 |
|  | 19292.8950 | .038 | .013 |
|  | 19514.6500 | .031 | .013 |
|  | 19615.3850 | .031 | .006 |
|  | 20083.8950 | .025 | .006 |
|  | 20856.2000 | .019 | .006 |
|  | 21761.9650 | .013 | .006 |
|  | 22333.0650 | .013 | .000 |
|  | 23024.7450 | .006 | .000 |
|  | 23706.7300 | .000 | .000 |
| Volume Right | 6185.6700 | 1.000 | 1.000 |
|  | 6207.3350 | .994 | 1.000 |
|  | 6735.2400 | .987 | 1.000 |
|  | 7355.2100 | .987 | .994 |
|  | 7522.0050 | .981 | .994 |
|  | 7578.5400 | .981 | .987 |
|  | 7622.4550 | .975 | .987 |
|  | 7751.4900 | .969 | .987 |
|  | 7841.4400 | .969 | .981 |
|  | 7928.7350 | .962 | .981 |
|  | 8051.4500 | .956 | .981 |
|  | 8209.3400 | .950 | .981 |
|  | 8330.6400 | .950 | .975 |
|  | 8364.5900 | .950 | .969 |
|  | 8425.6150 | .950 | .962 |
|  | 8508.9550 | .950 | .956 |
|  | 8575.6950 | .943 | .956 |
|  | 8680.8600 | .943 | .950 |
|  | 8775.6650 | .943 | .943 |
|  | 8796.3650 | .943 | .937 |
|  | 8841.0100 | .937 | .937 |
|  | 8872.6500 | .937 | .931 |
|  | 8919.6300 | .931 | .931 |
|  | 8983.3100 | .931 | .925 |
|  | 9009.8800 | .931 | .918 |
|  | 9014.9650 | .931 | .912 |
|  | 9036.2700 | .925 | .912 |
|  | 9056.1850 | .918 | .912 |
|  | 9101.4150 | .912 | .912 |
|  | 9155.7900 | .912 | .906 |
|  | 9169.6800 | .906 | .906 |
|  | 9174.7100 | .899 | .906 |
|  | 9206.8300 | .893 | .906 |
|  | 9239.5050 | .893 | .899 |
|  | 9242.2600 | .893 | .893 |
|  | 9246.7100 | .887 | .893 |
|  | 9266.9350 | .881 | .893 |
|  | 9285.3500 | .874 | .893 |
|  | 9294.3350 | .874 | .887 |
|  | 9336.8150 | .874 | .881 |
|  | 9385.0700 | .874 | .874 |
|  | 9401.8150 | .874 | .868 |
|  | 9433.4550 | .874 | .862 |
|  | 9466.8250 | .868 | .862 |
|  | 9472.4550 | .862 | .862 |
|  | 9475.0350 | .855 | .862 |
|  | 9477.6700 | .855 | .855 |
|  | 9488.1500 | .849 | .855 |
|  | 9513.3850 | .843 | .855 |
|  | 9541.2350 | .843 | .849 |
|  | 9562.5950 | .843 | .843 |
|  | 9572.2950 | .843 | .836 |
|  | 9603.0700 | .843 | .830 |
|  | 9639.3950 | .836 | .830 |
|  | 9655.1000 | .836 | .824 |
|  | 9674.8600 | .830 | .824 |
|  | 9687.0800 | .830 | .818 |
|  | 9721.8100 | .824 | .818 |
|  | 9766.7850 | .818 | .818 |
|  | 9779.2500 | .818 | .811 |
|  | 9783.7850 | .818 | .805 |
|  | 9790.2200 | .818 | .799 |
|  | 9804.0450 | .811 | .799 |
|  | 9831.4400 | .811 | .792 |
|  | 9848.1550 | .811 | .786 |
|  | 9856.2500 | .811 | .780 |
|  | 9876.9850 | .805 | .780 |
|  | 9905.8700 | .805 | .774 |
|  | 9936.2200 | .805 | .767 |
|  | 9954.9700 | .805 | .761 |
|  | 9970.5450 | .799 | .761 |
|  | 10001.9050 | .792 | .761 |
|  | 10032.6550 | .792 | .755 |
|  | 10057.3750 | .786 | .755 |
|  | 10075.2100 | .780 | .755 |
|  | 10097.3850 | .780 | .748 |
|  | 10120.7100 | .780 | .742 |
|  | 10136.2800 | .774 | .742 |
|  | 10169.1050 | .767 | .742 |
|  | 10204.2450 | .767 | .736 |
|  | 10223.2650 | .767 | .730 |
|  | 10230.7950 | .761 | .730 |
|  | 10241.0250 | .755 | .730 |
|  | 10250.9350 | .755 | .723 |
|  | 10263.8300 | .748 | .723 |
|  | 10291.5450 | .748 | .717 |
|  | 10315.9250 | .742 | .717 |
|  | 10325.6700 | .742 | .711 |
|  | 10329.0450 | .736 | .711 |
|  | 10347.9300 | .736 | .704 |
|  | 10373.1650 | .736 | .698 |
|  | 10399.5800 | .736 | .692 |
|  | 10434.0300 | .736 | .686 |
|  | 10457.9500 | .736 | .679 |
|  | 10472.2600 | .736 | .673 |
|  | 10483.8600 | .730 | .673 |
|  | 10488.2500 | .730 | .667 |
|  | 10489.7350 | .730 | .660 |
|  | 10498.1150 | .730 | .654 |
|  | 10515.4800 | .730 | .648 |
|  | 10530.1700 | .723 | .648 |
|  | 10538.1000 | .717 | .648 |
|  | 10543.3300 | .717 | .642 |
|  | 10549.6600 | .711 | .642 |
|  | 10560.5950 | .711 | .635 |
|  | 10571.7850 | .704 | .635 |
|  | 10576.7300 | .698 | .635 |
|  | 10578.8600 | .698 | .629 |
|  | 10583.1900 | .698 | .623 |
|  | 10587.0150 | .692 | .623 |
|  | 10591.6250 | .692 | .616 |
|  | 10609.2900 | .686 | .616 |
|  | 10627.8750 | .686 | .610 |
|  | 10642.3100 | .686 | .604 |
|  | 10660.3800 | .679 | .604 |
|  | 10673.8400 | .673 | .604 |
|  | 10683.9250 | .673 | .597 |
|  | 10714.6900 | .673 | .591 |
|  | 10750.0450 | .667 | .591 |
|  | 10762.7900 | .660 | .591 |
|  | 10766.7750 | .654 | .591 |
|  | 10774.1350 | .648 | .591 |
|  | 10809.3550 | .648 | .585 |
|  | 10849.3850 | .642 | .585 |
|  | 10862.8250 | .642 | .579 |
|  | 10865.2600 | .642 | .572 |
|  | 10866.2300 | .635 | .572 |
|  | 10876.7950 | .635 | .566 |
|  | 10896.9100 | .635 | .560 |
|  | 10911.9950 | .635 | .553 |
|  | 10919.4700 | .635 | .547 |
|  | 10924.1100 | .635 | .541 |
|  | 10935.5550 | .635 | .535 |
|  | 10950.6750 | .635 | .528 |
|  | 10960.3950 | .635 | .522 |
|  | 10964.5650 | .635 | .516 |
|  | 10985.4300 | .635 | .509 |
|  | 11019.3900 | .635 | .503 |
|  | 11039.0000 | .635 | .497 |
|  | 11060.1450 | .635 | .491 |
|  | 11087.3550 | .635 | .484 |
|  | 11120.6900 | .629 | .484 |
|  | 11150.0550 | .623 | .484 |
|  | 11161.2100 | .616 | .484 |
|  | 11175.1750 | .616 | .478 |
|  | 11188.9200 | .610 | .478 |
|  | 11192.9350 | .604 | .478 |
|  | 11194.4150 | .604 | .472 |
|  | 11198.0950 | .604 | .465 |
|  | 11219.2050 | .604 | .459 |
|  | 11253.3650 | .604 | .453 |
|  | 11270.4600 | .597 | .453 |
|  | 11272.5750 | .591 | .453 |
|  | 11279.8600 | .591 | .447 |
|  | 11298.2850 | .585 | .447 |
|  | 11329.8950 | .579 | .447 |
|  | 11349.0650 | .579 | .440 |
|  | 11349.7050 | .579 | .434 |
|  | 11370.7800 | .579 | .428 |
|  | 11392.7150 | .572 | .428 |
|  | 11398.2250 | .572 | .421 |
|  | 11425.5100 | .566 | .421 |
|  | 11449.2350 | .566 | .415 |
|  | 11449.8150 | .560 | .415 |
|  | 11456.1200 | .553 | .415 |
|  | 11474.7400 | .547 | .415 |
|  | 11495.5550 | .547 | .409 |
|  | 11506.0150 | .547 | .403 |
|  | 11511.7150 | .547 | .396 |
|  | 11518.1550 | .547 | .390 |
|  | 11536.1700 | .547 | .384 |
|  | 11558.6050 | .541 | .384 |
|  | 11571.9950 | .535 | .384 |
|  | 11587.3950 | .535 | .377 |
|  | 11617.5000 | .528 | .377 |
|  | 11639.0550 | .522 | .377 |
|  | 11664.7800 | .522 | .371 |
|  | 11690.4100 | .516 | .371 |
|  | 11699.3550 | .516 | .365 |
|  | 11717.4500 | .509 | .365 |
|  | 11730.5600 | .509 | .358 |
|  | 11741.6850 | .509 | .352 |
|  | 11760.5600 | .509 | .346 |
|  | 11794.2900 | .503 | .346 |
|  | 11842.2350 | .497 | .346 |
|  | 11873.4100 | .497 | .340 |
|  | 11886.4050 | .497 | .333 |
|  | 11896.6700 | .497 | .327 |
|  | 11925.9700 | .491 | .321 |
|  | 11967.6150 | .491 | .314 |
|  | 11994.6050 | .484 | .314 |
|  | 12011.4350 | .478 | .314 |
|  | 12019.5250 | .472 | .314 |
|  | 12025.0800 | .465 | .314 |
|  | 12030.5500 | .465 | .308 |
|  | 12040.4200 | .459 | .308 |
|  | 12054.4100 | .453 | .308 |
|  | 12064.8450 | .453 | .302 |
|  | 12075.4650 | .453 | .296 |
|  | 12091.5800 | .447 | .296 |
|  | 12103.0050 | .440 | .296 |
|  | 12126.5500 | .434 | .296 |
|  | 12150.7650 | .434 | .289 |
|  | 12155.0950 | .428 | .289 |
|  | 12162.6550 | .428 | .283 |
|  | 12200.7900 | .428 | .277 |
|  | 12234.4200 | .428 | .270 |
|  | 12240.3900 | .421 | .270 |
|  | 12257.1200 | .415 | .270 |
|  | 12304.5350 | .409 | .270 |
|  | 12344.7900 | .403 | .270 |
|  | 12358.8450 | .403 | .264 |
|  | 12373.5050 | .396 | .264 |
|  | 12384.9750 | .396 | .258 |
|  | 12392.1900 | .390 | .258 |
|  | 12395.8450 | .390 | .252 |
|  | 12410.0400 | .390 | .245 |
|  | 12422.3500 | .384 | .245 |
|  | 12431.0500 | .377 | .245 |
|  | 12454.3050 | .371 | .245 |
|  | 12469.6950 | .371 | .239 |
|  | 12471.3300 | .371 | .233 |
|  | 12482.8950 | .365 | .233 |
|  | 12495.4150 | .365 | .226 |
|  | 12504.7700 | .358 | .226 |
|  | 12529.7900 | .358 | .220 |
|  | 12554.1500 | .358 | .214 |
|  | 12563.9350 | .358 | .208 |
|  | 12573.2850 | .358 | .201 |
|  | 12588.2350 | .352 | .201 |
|  | 12628.4800 | .346 | .201 |
|  | 12675.6000 | .346 | .195 |
|  | 12714.8350 | .340 | .195 |
|  | 12740.2150 | .333 | .195 |
|  | 12753.6650 | .333 | .189 |
|  | 12778.6100 | .333 | .182 |
|  | 12794.8800 | .333 | .176 |
|  | 12820.8600 | .327 | .176 |
|  | 12855.1150 | .321 | .176 |
|  | 12868.4250 | .314 | .176 |
|  | 12880.9900 | .308 | .176 |
|  | 12894.6800 | .308 | .170 |
|  | 12898.9100 | .302 | .170 |
|  | 12919.7550 | .302 | .164 |
|  | 12939.8650 | .302 | .157 |
|  | 12981.0050 | .296 | .157 |
|  | 13033.7250 | .296 | .151 |
|  | 13064.1500 | .289 | .151 |
|  | 13101.8200 | .289 | .145 |
|  | 13144.4300 | .289 | .138 |
|  | 13176.0500 | .283 | .138 |
|  | 13186.5750 | .283 | .132 |
|  | 13224.4600 | .277 | .132 |
|  | 13265.6950 | .277 | .126 |
|  | 13272.9950 | .277 | .119 |
|  | 13287.2650 | .277 | .113 |
|  | 13311.9150 | .270 | .113 |
|  | 13325.2800 | .264 | .113 |
|  | 13338.7400 | .258 | .113 |
|  | 13358.7150 | .252 | .113 |
|  | 13405.3150 | .252 | .107 |
|  | 13457.5100 | .245 | .107 |
|  | 13486.7800 | .245 | .101 |
|  | 13536.9550 | .245 | .094 |
|  | 13585.5000 | .239 | .094 |
|  | 13644.6150 | .233 | .094 |
|  | 13701.3150 | .233 | .088 |
|  | 13750.2200 | .233 | .082 |
|  | 13787.5050 | .226 | .082 |
|  | 13789.5200 | .220 | .082 |
|  | 13817.4950 | .220 | .075 |
|  | 13849.1900 | .214 | .075 |
|  | 13871.0050 | .214 | .069 |
|  | 13894.5150 | .208 | .069 |
|  | 13901.8200 | .201 | .069 |
|  | 13904.9900 | .195 | .069 |
|  | 13959.5550 | .189 | .069 |
|  | 14014.1600 | .182 | .069 |
|  | 14037.6500 | .182 | .063 |
|  | 14068.2500 | .182 | .057 |
|  | 14112.0050 | .176 | .057 |
|  | 14163.9750 | .170 | .057 |
|  | 14194.6150 | .164 | .057 |
|  | 14242.7550 | .164 | .050 |
|  | 14289.2850 | .157 | .050 |
|  | 14309.0450 | .151 | .050 |
|  | 14320.7700 | .145 | .050 |
|  | 14345.4900 | .138 | .050 |
|  | 14436.3000 | .132 | .050 |
|  | 14543.0450 | .126 | .050 |
|  | 14581.2150 | .126 | .044 |
|  | 14600.6500 | .126 | .038 |
|  | 14708.7550 | .126 | .031 |
|  | 14834.1000 | .119 | .031 |
|  | 14884.3550 | .113 | .031 |
|  | 14922.2900 | .107 | .031 |
|  | 15020.4550 | .101 | .031 |
|  | 15102.8750 | .101 | .025 |
|  | 15120.1650 | .094 | .025 |
|  | 15148.0200 | .088 | .025 |
|  | 15169.6850 | .082 | .025 |
|  | 15194.1400 | .075 | .025 |
|  | 15274.0400 | .069 | .025 |
|  | 15361.8050 | .063 | .025 |
|  | 15447.7700 | .057 | .025 |
|  | 15513.9100 | .057 | .019 |
|  | 15764.3750 | .057 | .013 |
|  | 16011.6000 | .057 | .006 |
|  | 16040.2650 | .050 | .006 |
|  | 16084.9800 | .044 | .006 |
|  | 16240.5200 | .038 | .006 |
|  | 16708.2350 | .031 | .006 |
|  | 17276.5550 | .025 | .006 |
|  | 17696.9500 | .019 | .006 |
|  | 18007.1300 | .013 | .006 |
|  | 18277.5700 | .006 | .006 |
|  | 19024.6050 | .006 | .000 |
|  | 19625.4700 | .000 | .000 |
| The test result variable(s): Length Right, Width Left, Volume Right has at least one tie between the positive actual state group and the negative actual state group. | | | |
